# High hemoglobin level is a risk factor for maternal and fetal outcomes of pregnancy in Chinese women: A retrospective cohort study

**DOI:** 10.1186/s12884-022-04636-9

**Published:** 2022-04-06

**Authors:** Lanlan Wu, Ruifang Sun, Yao Liu, Zengyou Liu, Hengying Chen, Siwen Shen, Yuanhuan Wei, Guifang Deng

**Affiliations:** 1 Department of Clinical Nutrition, Union Shenzhen Hospital of Huazhong University of Science and Technology, No. 89 Taoyuan Road, Shenzhen, Guangdong 518052 P.R. China; 2 Department of Obstetrics, Union Shenzhen Hospital of Huazhong University of Science and Technology, Shenzhen, China; 3 Injury Prevention Research Center, Shantou University Medical College, Shantou, China

**Keywords:** Hemoglobin, Adverse pregnancy outcomes, Preterm birth, Low–birth–weight infants, Small–for–gestational–age infants

## Abstract

**Background:**

To examine the association of hemoglobin (Hb) levels during gestation with the risk of selected adverse pregnancy outcomes such as preterm birth (PTB), low-birth-weight infants (LBW) and small-for-gestational-age infants (SGA) in Chinese women.

**Methods:**

This retrospective cohort study was conducted in the Department of Gynecology and Obstetrics at the Union Shenzhen Hospital of the Huazhong University of Science and Technology, using routinely collected maternity and hospital data on pregnancies (2015–2018). Hb levels were measured during the second (16–18th weeks) and third (28–30th weeks) trimesters of pregnancy, and pregnancy outcomes were recorded in the hospital information system. Hb levels were categorized into four groups as follows: < 110 g/L, 110–119 g/L, 120–130 g/L, and > 130 g/L. The second group (Hb 110–119 g/L) was defined as the reference group. Statistical analysis was performed using multivariate logistic regression.

**Results:**

A total of 1911 singleton mothers were included. After multivariable adjustment, Hb levels > 130 g/L in the second trimester increased the risk of LBW (odds ratio [OR], 2.54; 95% confidence interval [CI], 1.12–5.76). In the third trimester of gestation, compared with women whose Hb levels between 110 and 119 g/L, women with Hb levels > 130 g/L had an increased risk of LBW (OR, 2.20; 95% CI, 1.07–4.51) and SGA (OR, 2.00; 95% CI, 1.05–3.80). When we compared the highest and lowest quartiles of changes in the Hb across the second and third trimesters, the adjusted ORs were 0.35 (95% CI: 0.18–0.68) for PTB and 0.47 (95% CI: 0.23–0.98) for LBW.

**Conclusion:**

Maternal Hb > 130 g/L was associated with increased risk of adverse pregnancy outcomes. Reduction of the risks of PTB and SGA were observed with the appropriate increase of Hb level during the third trimester.

## Background

The term “adverse pregnancy outcomes” refers to all pathological pregnancy and delivery complications, which not only significantly affect the physical and mental health of pregnant women and growing fetus but also directly threatens the lives of both mothers and new born [1]. The association of maternal hemoglobin (Hb) concentrations and pregnancy outcomes has been widely recognized by the public [2]. Although many studies have explored the relationship between anemia or high Hb and pregnancy outcomes, the research results have been inconsistent. In both low–income and high–income countries, anemia during pregnancy is one of the main risk factors for maternal death and adverse birth outcomes [3, 4]. While a large amount of studies have extensively documented the fact that anemia could cause a series of adverse maternal and birth outcomes, such as LBW, PTB, SGA, stillbirth, perinatal and neonatal death [3–9], a few studies has shown no correlation [10, 11]. It is generally accepted that pregnant women with moderate to severe anemia are at higher risk of adverse outcomes comparing to mild anemia [12, 13].

Compared with anemia, elevated maternal Hb levels are usually considered a predictor of well nutritional status and have not received enough attention. In recent decades, some studies have found that elevated Hb levels are associated with adverse maternal and infant outcomes, including gestational diabetes mellitus (GDM), PTB, LBW, SGA, and fetal death [2, 5, 8, 14], while a few studies has reported that high Hb levels are not associated with pregnancy outcomes [15]. In the United States, Switzerland and other developed countries, a U–shaped relationship between Hb concentrations and pregnancy outcomes has been found; that is, women with Hb values at either extreme have a higher risk of adverse birth outcomes [8]. However, the data from developing countries is relatively limited.

In addition, recent reviews have shown that the relationship between Hb levels and adverse pregnancy events is mainly affected by the time point of Hb determination [2]. Some studies have shown that anemia in first–trimester was significantly related to adverse pregnancy outcomes [5, 16, 17], while other studies have shown that this relationship was more significant in the second or third trimesters [18]. Furthermore, there have been few studies on the changes of Hb levels between the second and third trimester of gestation on maternal and infant pregnancy outcomes.

Interestingly, previous studies have only measured Hb concentrations at single time points and have few tracked changes over time during pregnancy. By collecting blood samples across multiple time points, one can assess longitudinal changes and thereby perform a more robust analysis of the correlations between the concentration of Hb and the risk of developing adverse pregnancy outcomes. In this retrospective cohort study, we aimed to examine the association of Hb levels in the second and third trimesters of pregnancy with adverse pregnancy outcomes through the detection of Hb levels at different stages of pregnancy in Chinese women and to evaluate the influence of the changes of Hb levels between the second and third trimester of gestation on maternal and infant pregnancy outcomes.

## Methods

### Study design and participants

We have not performed a sample size assessment before planning the study, and the retrospective study included all the pregnant women in the Department of Gynecology and Obstetrics at the Union Shenzhen Hospital of the Huazhong University of Science and Technology (Shenzhen, Guangdong) from January 2015 to December 2018. The inclusion criteria of subjects were as follows: 1) registered and planned to deliver their child at this hospital; 2) singleton pregnancy; 3) had complete medical records. Then a total of 1994 pregnant women were recruited in the study. From the time of the first prenatal examination to the time of delivery, all clinical characteristics and biochemical indicators of the participants were recorded in the hospital information system. Participants were excluded, as they had one of the following conditions: smoking or drinking alcohol during pregnancy (*n* = 4), history of liver disease (*n* = 12), diabetes or hypertension (*n* = 45), kidney disease (*n* = 5), heart disease (*n* = 3), and twin or multiple pregnancy (*n* = 14). Finally, a total of 1911 gravidas were included in the present investigation. Informed consent was obtained from all subjects and/or their legal guardians, and all procedures were approved by the Ethics Committee of the Union Shenzhen Hospital of Huazhong University of Science and Technology.

### Baseline information and potential confounders

Age (years), education (primary, secondary, college or above), conception method (natural or artificial)**,** parity (nulliparity or multipara)**,** history of miscarriage (yes or no), multiple or singleton pregnancy and history of disease (e.g., liver disease, diabetes or hypertension, kidney disease, heart disease) were obtained through face-to-face interviews by a well-trained investigator, and questionnaires were completed simultaneously. Height and weight were measured at 16–18 weeks of pregnancy using an electronic scale with the following detailed instructions: take off the shoes, stand up straight with the shoulders parallel and the body naturally relaxed. Height and weight measurements were accurate to 0.1 cm and 0.1 kg, respectively. The body mass index (BMI) (kg/m^2^) at 16–18 weeks was calculated as weight (kg) divided by the square of the height (m^2^).

Based on the diagnostic criteria of the International Association of Diabetes and Pregnancy Study Group (IADPSG), all women were screened for GDM during the 24–28 week of gestation. GDM was diagnosed if the fasting plasma glucose levels reached 5.1 mmol/L or if glucose levels reached 10 mmol/L within 1 h or 8.5 mmol/L within 2 h of an oral glucose tolerance test (OGTT) [19]. OGTT was performed using a one-step method during the 24–28th week of pregnancy.

Generally speaking, when the Hb level of Chinese pregnant women is less than 110 g/L, the treatment method will be selected according to the degree of anemia. Mild to moderate anemia is mainly treated with oral iron (supplement elemental iron 100 ~ 200 mg/d) and improve the structure of dietary; Pregnant women with severe anemia are treated with oral iron or injection of iron. All interventions are carried out under unified guidelines and will not affect the results.

### Laboratory assays

At 16–18 weeks and 28–30 weeks of pregnancy, fasting venous blood samples were collected by a professionally trained investigator. Since the 1990s, serum marker screening for fetal Down syndrome was introduced into China, and the more commonly adopted program is to screen pregnant women for Down syndrome in the second-trimester, and a few pregnant women in the first-trimester. 95% of samples for down syndrome were collected between 15 and 19 weeks gestation. But 16–18 weeks is the best time for down syndrome screening, blood routine and urine routine are also required simultaneously, and thyroid function, fasting blood glucose, liver function and renal function may be tested. Therefore, pregnant women are usually advised to coming to the hospital on an empty stomach and take some food so that they can supplement nutrition in time after venous blood collection to prevent hypoglycemia. Hb levels were measured using an automated colorimetric procedure. All laboratory measurements were performed with a Hitachi 7600 automatic analyzer (Hitachi, Tokyo, Japan).

### Assessment of pregnancy outcomes

We followed the definition of the International Classification of Diseases, 10th Revision (ICD–10). However, ICD-10 codes were occasionally missing or wrong, so we combined the information such as gestational age and neonatal weight to determine the pregnancy outcomes. Inpatient medical charts were reviewed to verify clinical information. PTB was defined as delivery < 37 completed weeks of gestation (ICD-10 codes was P07.301, O60, O60.X01). Low birth weight was defined as a newborn with a birth weight less than 2500 g (ICD-10 codes was P05.051, P07.001) [20]. Infants were defined as SGA when their birth weights were below the 10th percentile on the growth chart (ICD-10 codes was P05.101) [21]. The growth curve was referred to the growth standard curves of birth weight, length and head circumference of Chinese newborns of different gestation based on 24,375 newborns with gestational age between 24 to 42 weeks in 9 cities of China published in 2020 [22].

### Statistical analyses

Baseline information is presented as the median (quartiles) for continuous variables and as the proportion (%) for categorical variables. Statistical differences between groups were tested using Kruskal-Wallis for continuous variables and the chi-square test for categorical variables. Hb levels were categorized into four groups as follows: < 110 g/L, 110–119 g/L, 120–130 g/L, and > 130 g/L. The second group (110 g/L ≤ Hb < 119 g/L) was defined as the reference group. Odds ratios (ORs) and 95% confidence intervals (95% CIs) were calculated by using logistic regression models to examine the association of Hb levels at gestation with the risk of adverse pregnancy outcomes. Two models were included in the present study: Model 1 was unadjusted; Model 2 was adjusted for age, BMI, education, conception method, parity, history of miscarriage and GDM. To explore the effect of changes in Hb concentration on pregnancy outcomes, D-value (D-value = Hb concentration in the 3rd trimester – Hb concentration in the 2nd trimester) were categorized by quartiles and logistic regression analysis was performed using the lowest quartile as the reference group for all participants. Furthermore, pregnant women with low Hb levels (< 110 g/L) or high Hb levels (> 130 g/L) at 16–18 weeks were divided into two groups: changed to 110–129 g/L and not according to Hb levels at 28–30 weeks. All analyses were carried out by using SPSS 24.0 (SPSS Inc., Chicago, IL, USA), and a two-sided *p-*value < 0.05 was considered statistically significant. Graphic production was completed by using R version 3.0.3 (The R Foundation for Statistical Computing, Vienna, Austria).

## Results

### Baseline characteristics

A total of 1911 singleton pregnant women aged 31.0 (29.0–34.0) years were included in the study with 108 cases of PTB, 66 cases of LBW and 86 cases of SGA. As presented in Table 1, the differences in BMI, conception method, prevalence of GDM, and Hb levels in the second and third trimesters of gestation were statistically significant (*p* < 0.05) among the four Hb levels. There was no significant difference in other indicators.

**Table 1:** Baseline characteristics of mothers and infants by hemoglobin (Hb) levels in the second trimester in this study

	Total	Hb levels (g/L)				*P* value
		< 110	110–119	120–129	> 130	
No. of maternal cases	1911	405	779	583	144	
Maternal age (years)	31.0 (29.0, 34.0)	31.0 (28.5, 35.0)	31.0 (29.0, 34.0)	31.0 (29.0, 34.0)	31.0 (29.0, 35.0)	0.874
Age categories						0.278
< 35	1445 (75.6)	297 (73.3)	592 (76.0)	451 (77.4)	105 (72.9)	
≥ 35	466 (24.4)	108 (26.7)	187 (24.0)	132 (22.6)	39 (27.1)	
BMI (kg/m^2^)		20.0 (18.4, 21.5)	20.5 (18.9, 21.9)	20.7 (19.1, 22.6)	21.7 (19.7, 23.6)	< 0.001
Education						0.493
Primary	78 (4.1)	20 (4.9)	27 (3.5)	24 (4.1)	7 (4.9)	
Secondary	285 (14.9)	68 (16.8)	122 (15.7)	78 (13.4)	17 (11.8)	
College or above	1548 (81.0)	317 (78.3)	630 (80.9)	481 (82.5)	120 (83.3)	
Conception method						0.015
Natural	1869 (97.8)	383 (94.6)	768 (98.6)	576 (98.8)	142 (98.6)	
Artificial	26 (1.4)	12 (3.0)	7 (0.9)	5 (0.9)	2 (1.2)	
Parity						0.113
Nulliparity	815 (42.6)	180 (44.4)	314 (40.3)	257 (44.1)	64 (44.4)	
Multipara	1096 (57.4)	225 (55.5)	465 (59.7)	326 (55.9)	80 (55.6)	
History of miscarriage	819 (42.9)	173 (42.7)	340 (43.6)	242 (41.5)	80 (55.6)	0.854
GDM	372 (19.5)	59 (14.6)	154 (19.8)	126 (21.6)	33 (22.9)	0.029
Hemoglobin (g/L)						
2nd trimester	117.0 (111.0, 123.0)	106.0 (102.0, 108.0)	115.0 (112.5, 117.3)	123.5 (121.5, 126.0)	133.0 (131.0, 135.0)	< 0.001
3rd trimester	118 (111.3, 124.3)	110.3 (105.3, 116.0)	117.5 (111.7, 122.5)	122.5 (116.3, 127.3)	128.2 (119.0, 134.5)	< 0.001
Adverse maternal and fetal outcomes						
PTB	108 (5.7)	23 (5.7)	44 (5.6)	31 (5.3)	10 (6.9)	0.902
LBW	66 (3.5)	15 (3.7)	21 (2.7)	21 (3.6)	9 (6.3)	0.184
SGA	86 (4.5)	24 (5.9)	31 (4.0)	24 (4.1)	7 (4.9)	0.449

Data are presented as the median (quartiles) for continuous variables and as n (%) for categorical variables

*Abbreviations*: *BMI* body mass index, *GDM* gestational diabetes mellitus, *LBW* low-birth-weight infants, *PTB* preterm birth, *SGA* small-for-gestational-age infants

### Association of Hb levels with adverse pregnancy outcomes

The associations of Hb levels in the second trimester with adverse pregnancy outcomes are shown in Table 2. The second trimester Hb levels were not significantly associated with PTB or SGA. However, mothers with Hb levels > 130 g/L had a 154% (95% CI, 1.12–5.76) higher risk of LBW than those with Hb levels of 110–119 g/L.

**Table 2:** ORs (95% CIs) for adverse pregnancy outcomes according to the hemoglobin (Hb) levels at the second trimester

	Hb levels (g/L)			
	< 110	110–119	120–129	> 130
PTB				
Case/N	23/405	44/779	31/583	10/144
Model 1	1.01 (0.60, 1.69)	1	0.94 (0.59, 1.51)	1.25 (0.61, 2.54)
Model 2	1.05 (0.62, 1.77)	1	0.95 (0.59, 1.54)	1.25 (0.61, 2.58)
LBW				
Case/N	15/405	21/779	21/583	9/144
Model 1	1.39 (0.71, 2.72)	1	1.35 (0.73, 2.49)	2.41 (1.08, 5.37)
Model 2	1.29 (0.65, 2.57)	1	1.39 (0.75, 2.59)	2.54 (1.12, 5.76)
SGA				
Case/N	24/405	31/779	24/583	7/144
Model 1	1.52 (0.88, 2.63)	1	1.04 (0.60, 1.79)	1.23 (0.53, 2.86)
Model 2	1.37 (0.78, 2.39)	1	1.08 (0.63, 3.40)	1.45 (0.62, 3.40)

Model 1: without adjustment

Model 2: adjustment for age, BMI, education, conception method, parity and history of miscarriage and GDM

*Abbreviations*: *PTB* preterm birth, *LBW* low-birth-weight infants, *SGA* small-for-gestational-age infants

The associations of Hb levels in the third trimester with adverse pregnancy outcomes are shown in Table 3. Compared with those for Hb levels of 110–119 g/L, the OR (95% CI) for the risk of LBW at high levels was 2.20 (1.07–4.51), and the OR (95% CI) of SGA at high levels was 2.00 (1.05–3.80).

**Table 3:** ORs (95% CIs) for adverse pregnancy outcomes according to hemoglobin (Hb) levels at the third trimester

	Hb levels (g/L)			
	< 110	110–119	120–129	> 130
PTB				
Case/N	29/392	37/698	25/609	17/212
Model 1	1.43 (0.86, 2.36)	1	0.77 (0.46, 1.29)	1.56 (0.86, 2.83)
Model 2	1.51 (0.91, 2.51)	1	0.79 (0.47, 1.34)	1.64 (0.89, 3.01)
LBW				
Case/N	16/392	22/698	15/609	6/212
Model 1	1.31 (0.68, 2.52)	1	0.78 (0.40, 1.51)	2.01 (0.99, 4.07)
Model 2	1.40 (0.72, 2.72)	1	0.82 (0.42, 1.60)	2.20 (1.07, 4.51)
SGA				
Case/N	18/392	29/698	23/609	16/212
Model 1	1.11 (0.61, 2.03)	1	0.91 (0.52, 1.58)	1.88 (1.01, 3.54)
Model 2	1.11 (0.60, 2.03)	1	0.94 (0.53, 1.65)	2.00 (1.05, 3.80)

Model 1: without adjustment

Model 2: adjustment for age, BMI, education, conception method, parity, history of miscarriage and GDM

*Abbreviations*: *PTB* preterm birth, *LBW* low-birth-weight infants, *SGA* small-for-gestational-age infants

### Association of the changes of Hb levels with adverse pregnancy outcomes

The associations of D-value for Hb levels with adverse pregnancy outcomes were shown in Table 4. Compared with those for D-value in Q1, the OR (95% CI) for the risk of PTB in Q4 was 0.35 (0.18–0.68), and the OR (95% CI) of LBW at Q4 was 0.47 (0.23–0.98).

**Table 4:** ORs (95% CIs) for adverse pregnancy outcomes according to the quartiles of D-value for hemoglobin (Hb) levels

	D-value for Hb levels (g/L)				*P*_*trend*_
	Q1 (<−4.49)	Q2 (−4.48–1.67)	Q3 (1.68–7.17)	Q4 (> 7.18)	
PTB					
Case/N	35/478	31/484	29/475	13/474	
Model 1	1	0.87 (0.53, 1.43)	0.82 (0.49, 1.37)	0.36 (0.19, 0.68)	0.003
Model 2	1	0.87 (0.53, 1.44)	0.80 (0.48, 1.35)	0.35 (0.18, 0.68)	0.003
LBW					
Case/N	23/478	13/484	18/475	12/474	
Model 1	1	0.55 (0.27, 1.09)	0.78 (0.42, 1.46)	0.52 (0.25, 1.05)	0.127
Model 2	1	0.54 (0.27, 1.09)	0.79 (0.42, 1.49)	0.47 (0.23, 0.98)	0.089
SGA					
Case/N	17/478	20/484	25/475	24/474	
Model 1	1	1.17 (0.61, 2.26)	1.51 (0.80, 2.83)	1.45 (0.77, 2.73)	0.184
Model 2	1	1.16 (0.60, 2.26)	1.45 (0.77, 2.74)	1.27 (0.66, 2.43)	0.371

Model 1: without adjustment

Model 2: adjustment for age, BMI, education, conception method, parity, history of miscarriage and GDM

*Abbreviations*: *PTB* preterm birth, *LBW* low-birth-weight infants, *SGA* small-for-gestational-age infants

**Fig. 1:**
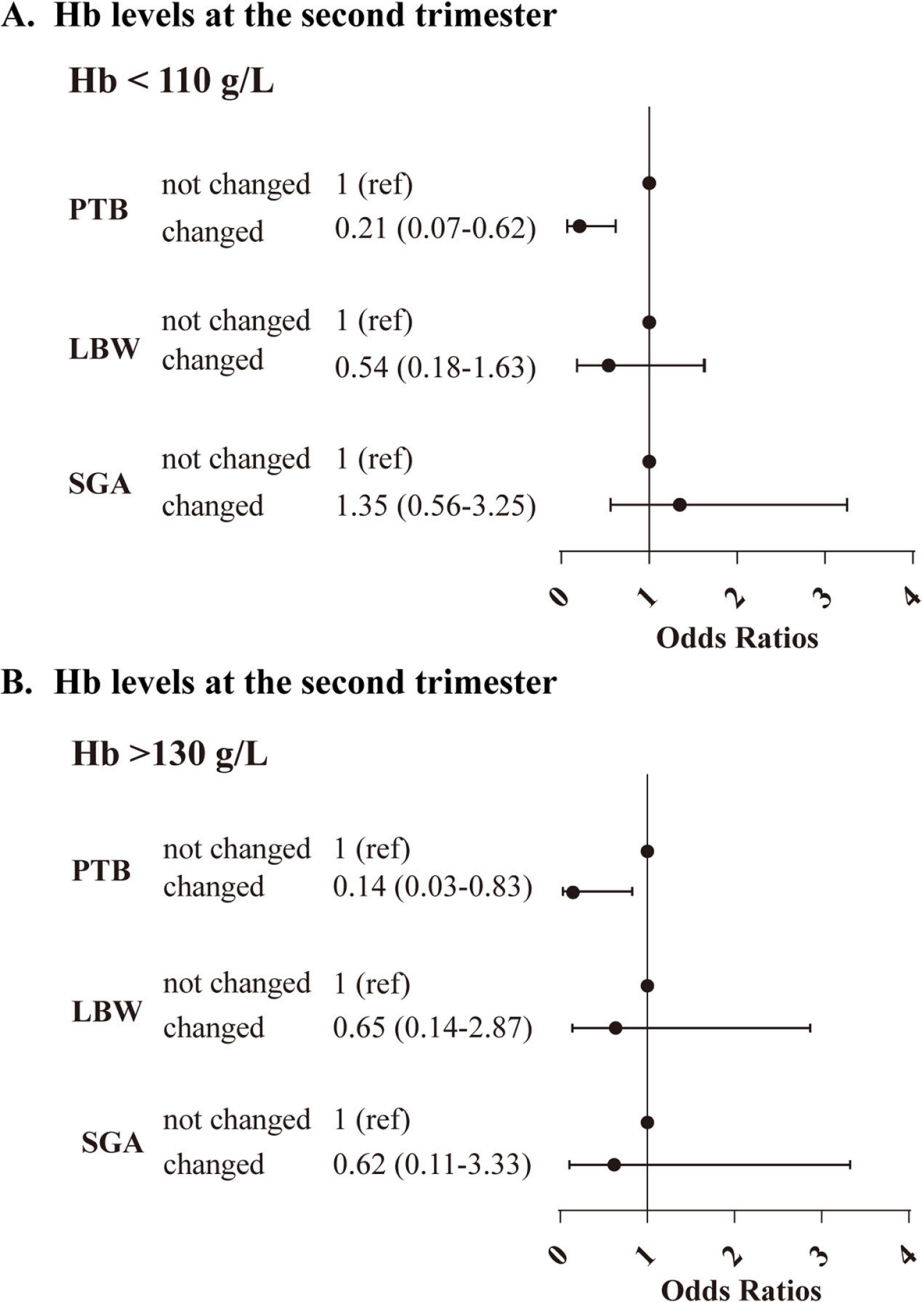
The risk of women whose Hb in the second trimester changed to 110–129 g/L in the third trimester vs not changed. Legends. The model was adjusted for age, prepregnancy BMI, education, conception method, number of pregnancies, parity, history of miscarriage and gestational diabetes

Figure 1 showed that women whose Hb < 110 g/L in the second trimester changed to 110–129 g/L in the third trimester vs not changed (reference) had a reduced risk of PTB (OR, 0.21; 95% CI, 0.07–0.62). The same results were found in women with Hb levels > 130 g/L in the second trimester (OR, 0.14; 95% CI, 0.0–0.83).

## Discussion

In this retrospective cohort study, we observed an increased risk of LBW in pregnant women with elevated Hb levels during the 16–18th weeks of gestation. During the 28–30th weeks of gestation, pregnant women with higher Hb levels exhibited an increased risk of LBW and SGA. In addition, mothers with moderately increased Hb levels during the third trimester of pregnancy had a reduced risk of PTB and LBW.

High levels of Hb during pregnancy are generally considered to be caused by failure of the plasma volume to expand, which increases maternal blood viscosity [23]. Hyperviscosity is negatively affected on the intervillous space, leading to poor maternal-fetal exchange [24]. This could directly cause fetal growth disorders through the reduction in nutrient transfer or may indirectly lead to fetal growth disorders through the release of fetal corticosteroids in response to chronic fetal hypoxia [23]. Recently, experimental studies have found that Hb levels have the ability to bind and inactivate nitric oxide (NO), which is an endothelial–derived smooth muscle relaxing factor [25]. With the increased hematocrit and decreased oxygen level, this ability becomes counterproductive [26]. The deactivation of NO then lead to oxidative stress, vasoconstriction and placental ischemia [27]. In addition, Magnus Centlow, Stefan R. Hansson and M Centlow suggested that fetal Hb at the placental level is overproduced and leaks into the maternal circulation, further damaging placental function [28–30]. More than a decade ago, both Georg Friedrich von Tempelhoff and Rasmussen found a negative correlation between Hb levels in the second trimester of pregnancy and birth weight [11]. In a recent study of 31,906 Australian pregnant women, Deborah A. Randall showed that pregnant women with a high concentration of Hb (≥ 140 g/L) had higher incidence of adverse events such as PTB and very low birth weight [31]. Similarly, Justiina Ronkainen et al. recently confirmed that high Hb levels (above the 90th percentile) in late pregnancy were associated with an increased risk of SGA (OR 1.60, 95% CI [1.26, 2.02]) in two Finnish birth cohorts [18]. In contrast, a population–based study in Chinese by A. Ren et al. reported that high Hb levels play no role in adverse pregnancy outcomes [15]. These findings from previous studies are inconsistent and there are few studies on the association between high Hb and adverse pregnancy outcomes among Chinese women. Previous studies have demonstrated that ethnicity potentially modifies the relationship between Hb and pregnancy outcomes [32]. We extended these findings in a relatively large cohort of pregnant Chinese women and observed that women with high Hb levels during the 16–18th week of pregnancy exhibited a 154% higher risk of LBW and during the 28–30th week of pregnancy exhibited a 120 and 100% higher risk of LBW and SGA, respectively. The heterogeneous nature of the results reported in previous studies may be due to variations in study design, sample size, the time points of Hb determination, the level of grading, or other confounding factors.

It has long been noted that anemia is a risk factor for various adverse maternal, fetal and neonatal outcomes. Low Hb levels could limit the oxygen circulation in the body, resulting in oxidative stress or chronic hypoxia, which could then cause adverse maternal and infant outcomes [16]. The WHO defines the Hb threshold for maternal anemia as follows: 100 to 110 g/L for mild anemia, 70 to 100 g/L for moderate, and 70 g/L for severe. But in the second trimester of pregnancy, the threshold of anemia was reduced to 105 g/L. A meta-analysis by Naoko Kozuki et al. suggested that mild anemia showed no significant relationship with SGA and that both the < 90 categories were associated with a 53% increase in the risk of SGA (OR = 1.53, 95% CI: 1.24–1.87) [13]. In another investigation, pregnant women with mild anemia with Hb concentrations of 8–10.9 g/L had no increased risk of PTB and LBW [12]. The majority of the pregnant women with Hb < 110 g/L in the second trimester were diagnosed with mild anemia, and there were few cases of moderate or severe anemia, which may be one of the reasons why low levels of Hb category in this study were not associated with adverse pregnancy outcomes. With the plasma volume increase during pregnancy, the Hb concentration continues to fall until the second trimester, then increases slightly around the 30th week. Therefore, measurements taken during or after the second trimester of pregnancy are likely to weaken the link between low Hb and adverse maternal and infant outcomes, which may be another reason for the current results.

One interesting finding from this study was that an elevated Hb concentration during the third trimester led to a decreased risk of developing PTB and SGA in a dose-dependent manner. These results highlight the importance of tracking Hb concentrations across the whole duration of pregnancy. Further analysis found that women with Hb levels < 110 g/L in the second trimester changed to 110–129 g/L vs not changed in the third trimester had a 79% reduced risk of PTB. The possible mechanism is that the increase of Hb levels in the third trimester reduces the risk of infections, resulting in the decrease of production of corticotrophin releasing hormone (CRH), elevated concentrations of which have been identified as a major risk factor of preterm birth. Another possible reason is that the improvement of low Hb levels during pregnancy alleviates the hypoxia and stress response caused by low Hb, thereby reducing the CRH released by the placenta and the secretion of cortisol associated with PTB [33]. We also found that women with Hb levels > 130 g/L in the second trimester had an 79% lower risk of PTB than women with no change in Hb levels in the third trimester. However, clinicians do not take steps to correct high hemoglobin levels in healthy women and this occurs usually by expansion of the plasma volume. Further longitudinal studies with larger sample sizes are needed to validate our findings. Although the specific guidelines varied in different countries, routine blood tests are generally recommended at each trimester of pregnancy. In China, to prevent iron deficiency anemia in pregnant women routine iron supplementation is usually started at the third month of pregnancy and blood tests are repeated every 8–12 weeks. Due to its low cost and relatively easy determination, hemoglobin concentration is often used as an alternative indicator of iron deficiency anemia. During the second trimester of pregnancy, the plasma expands further and the iron requirement increases. At this time, it is particularly important to recheck the blood routine to determine the hemoglobin concentration so that the next treatment plan can be made. Our findings may support shortening follow–up intervals and increasing follow-up times and timely adjustment of treatment plans based on changes in Hb levels in pregnant women at different times to minimize the risk of preterm delivery or altered fetal development.

Although our study comprehensively explored the association between maternal Hb concentrations and the risk of adverse pregnancy outcomes in a relatively large sample size, some limitations in this study remain. First, the small size of the subgroup of women aged > 35 years and with BMI > 24 kg/m^2^ limited the statistical power. Second, the analytic cohort was from China, which may limit the generalizability of the study results. Finally, although we accounted for known confounders, some unmeasured or unknown residual confounders remained (either unmeasured or unknown).

## Conclusions

High levels of Hb in the second and third trimesters of pregnancy increase the risk of adverse pregnancy outcomes. Reduction of the risks of PTB and SGA were observed with the appropriate increase of Hb level after the second trimester. Active treatment could help to reduce some adverse maternal and infant events, when abnormal Hb levels are found during mid-term pregnancy examinations.

## Data Availability

To protect the privacy of pregnant women, the datasets used and/or analyzed during the current study are available from the corresponding author on reasonable request.

## Abbreviations

BMI: Body mass index
CRH: Corticotrophin releasing hormone
GDM: Gestational diabetes mellitus
Hb: Hemoglobin
LBW: Low–birth–weight infants
NO: Nitric oxide
OR: Odds ratio
95% CIs: 95% confidence intervals
SGA: Small–for–gestational–age infants
PTB: Preterm birth
